# Venezuelan Equine Encephalitis and 2 Human Deaths, Peru

**DOI:** 10.3201/eid1603.090970

**Published:** 2010-03

**Authors:** Stalin Vilcarromero, Patricia V. Aguilar, Eric S. Halsey, Alberto Laguna-Torres, Hugo Razuri, Juan Perez, Yadira Valderrama, Eduardo Gotuzzo, Luis Suárez, Manuel Céspedes, Tadeusz J. Kochel

**Affiliations:** Naval Medical Research Center Detachment, Lima, Peru (S. Vilcarromero, P.V. Aguilar, E.S. Halsey, V.A. Laguna-Torres, H. Razuri, J. Perez, T.J. Kochel); Universidad Peruana Cayetano Heredia, Lima (S. Vilcarromero, E. Gotuzzo); Hospital de Apoyo Yurimaguas, Loreto, Peru (Y. Valderrama); Dirección General de Epidemiología, Lima (L. Suárez); Instituto Nacional de Salud, Lima (M. Céspedes)

**Keywords:** Venezuelan equine encephalitis, VEE, encephalitis, neurological disease, Panama/Peru genotype, Amazon Basin, viruses, zoonoses, Peru, dispatch

## Abstract

Studies have suggested that enzootic strains of Venezuelan equine encephalitis (VEE) subtype ID in the Amazon region, Peru, may be less pathogenic to humans than are epizootic variants. Deaths of 2 persons with evidence of acute VEE virus infection indicate that fatal VEEV infection in Peru is likely. Cases may remain underreported.

Venezuelan equine encephalitis (VEE) is an emerging zoonotic disease in the Amazon region of Peru. After dengue, it is considered the second most important arboviral disease in Peru. Most human infections with VEE virus (VEEV) are caused by subtype ID (*1*–*5*), and within subtype ID, 6 genotypes have been described (*6*). In Peru, the Colombia/Venezuela, Panama/Peru, and Peru/Bolivia genotypes have been identified among VEEV subtype ID isolates (*1*,*5**,**7*). Epidemiologic investigations have failed to detect neurologic disease or deaths among >200 VEE cases in this country (T.J. Kochel, unpub. data). Only 2 fatal cases with VEEV subtype ID have been reported, both in Panama (*6**,**8*). In contrast, fatal cases with neurologic complications (estimated mortality rate 0.7%) have been described regularly for human outbreaks caused by VEEV subtypes IAB and IC (*9*–*12*). On the basis of these reports, it has been suggested that enzootic VEEV strains in Peru may be less pathogenic to humans than the epizootic variants (*13*). However, only 200 cases identified in Peru may not be enough to make such an assertion.

We recently described a severe infection in a 3-year-old boy who had VEEV subtype ID (*14*). Here we describe 2 fatal infections in persons with evidence of acute VEEV infection in Peru. One patient had confirmed subtype ID.

## The Study

In 2000, the Naval Medical Research Center Detachment (NMRCD) and the Ministry of Health of Peru established a passive surveillance study to determine arboviral causes of febrile illness (protocol NMRCD.2000.0006). Patients with acute, undifferentiated febrile illness of <7 days were invited to enroll, and demographic and clinical information was obtained at the time of enrollment. Blood samples were obtained and assayed by virus isolation, and convalescent-phase samples were obtained 10 days to 4 weeks later for serologic studies.

**Figure 1:**
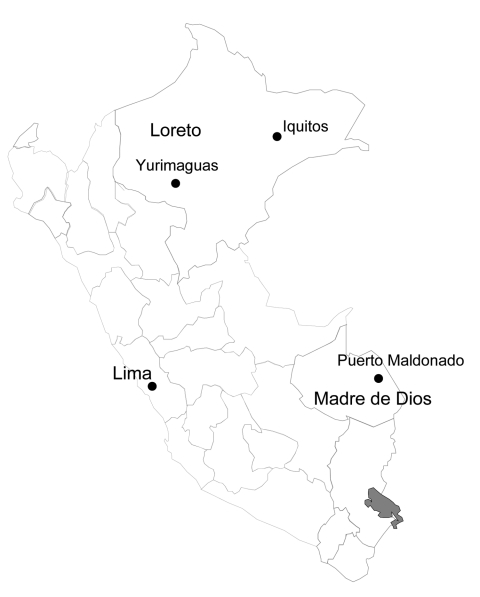
Selected sites in Peru of passive surveillance study to determine arboviral causes of febrile illness in Peru, established in 2000 by Naval Medical Research Center Detachment and the Ministry of Health of Peru (protocol NMRCD.2000.0006). Sites shown include those of 2 patients with evidence of acute Venezuelan equine encephalitis virus infection. Shaded area is Titicaca Lake.

Patient 1 was a 7-year-old girl from San Benito, a rural community near Yurimaguas city (Figure 1), who on June 19, 2006, was noted to have nasal congestion, sneezing, chills, malaise, myalgia, abdominal pain, and fever followed by several episodes of watery, nonbloody feces, and vomiting. Her condition rapidly worsened, and she began having tonic–clonic seizures. The next day the involuntary movement and vomiting stopped; however, other signs worsened and she became somnolent and prostrate. Later that day she was admitted to the Hospital Santa Gema of Yurimaguas with fever, dehydration, stupor, and signs of respiratory distress along with tonic–clonic seizures. Temperature was 40°C (104°F), blood pressure 90/50 mm Hg, respiratory rate markedly increased, and heart rate 132 beats/min. Distal cyanosis, nasal flaring, rhonchi without crackles, and supraclavicular and intercostal retractions were noted, but no jaundice, lymphadenopathy or conjunctival hemorrhage were observed. Hepatomegaly (liver 4 cm under the costal border), stupor, and neck stiffness were also noted. Laboratory test results showed a left shift in the leukocyte count and renal failure (Table 1). Blood smears were negative for malaria parasites. Preliminary diagnoses were sepsis, convulsive status, and respiratory distress. Later that day the patient was considered to have a complicated, febrile, neurologic illness, and a blood sample was sent for advanced analysis at the NMRCD in Lima, Peru, and the National Institute of Health of Peru.

**Table 1:** Laboratory test results for 2 patients infected with Venezuelan equine encephalitis virus, Peru*

Testing	Patient no. 1		Patient no. 2		Reference range
Date blood collected	2006 Jun 20	2005 Feb 28	2005 Mar 1	
Hematocrit, %	36		36	22	38–44
Thromocytes, cells/mm^3^	233,000		143,750	NM	150,000–450,000
Leukocytes					
Total, cells/mm^3^	21,450		3,900	NM	4,500–10,000
Bands, %	7		4	NM	3–5
Segmented cells, %	84		72	NM	55–65
Eosinophils, %	0		0	NM	0.5–4.0
Monocytes, %	0		14	NM	3–8
Lymphocytes, %	9		10	NM	25–35
ESR, mm/h	41		NM	NM	1–15
Creatinine, mg/dL	2.1		9.1	8.8	0.8–1.4
Urea, mg/dL	115		206	238	20–45

*NM, not measured; ESR, erythrocyte sedimentation rate.

The girl was given supportive therapy with broad spectrum antibiotics, intravenous hydration, oxygen, and anticonvulsive medications (diazepam, phenytoin). On June 21, seizures persisted, and the patient became comatose and later died of respiratory arrest. Arterial blood gasses and electrolytes could not be measured because of the limited capacity of the hospital laboratory. The girl’s parents did not consent to lumbar puncture or autopsy.

**Figure 2:**
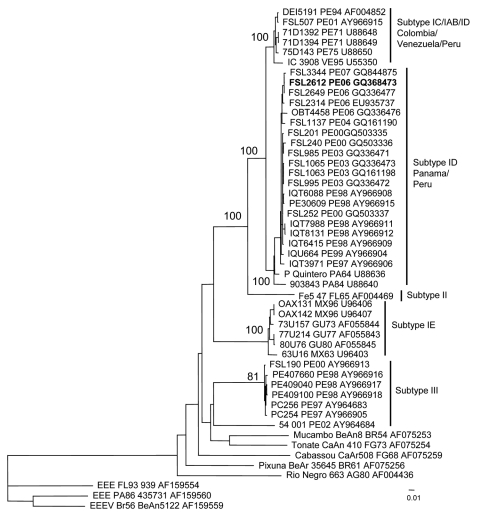
Neighbor-joining phylogenetic tree of Venezuelan equine encephalitis virus (VEEV) complex based on partial sequence of the PE2 segment (nucleotide positions ≈8385–9190 of the VEEV genome). The tree was rooted by using an outgroup of 3 major lineages of Eastern equine encephalitis virus (EEEV). The strain isolated from a 7-year-old girl who died from acute VEEV infection in Peru, June 21, 2006, is in **boldface.** Viruses are labeled by code designation, abbreviated location name, year of isolation (last 2 digits of year only), and GenBank accession numbers of the corresponding sequences. PA, Panama; GU, Guatemala; MX, Mexico; FG, French Guiana; VE, Venezuela; BR, Brazil; AG, Argentina; PE, Peru; FL, Florida. Numbers indicate bootstrap values. Scale bar indicates nucleotide substitutions per site.

Examination of the girl’s serum in Vero cells identified VEEV, and sequencing and phylogenetic analyses using previously described methods further identified it as subtype ID Panama/Peru genotype (Figure 2) (*15*). On the day the serum was collected (1 day after onset of signs), viremia titer was 1.8 ×10^4^ PFU/mL, similar to titers from other VEEV-infected study patients who did not have neurologic complications (Table 2). The National Institute of Health reported the sample to be negative for leptospiral and rickettsial organisms, according to ELISA immunoglobulin (Ig) M and indirect immunofluorescent assays, respectively. Blood and cerebrospinal fluid cultures for bacteria were not attempted.

**Table 2:** Venezuelan equine encephalitis virus titers in 10 patients, Peru

Patient code	Age, y/sex	Location	Date of symptom onset	Date of blood collection	Serum titer, PFU/mL	Signs and symptoms						
						Death	Bleed*	Fever	Seizure	GI†	Resp‡	Aches§
Patient 1 FSL2612	7/F	Yurimaguas, Loreto	2006 Jun 19	2006 Jun 20	1.8 × 10^4^	+	–	+	+	+	+	+
FSL2649	32/M	Yurimaguas, Loreto	2006 Aug 7	2006 Aug 8	3.4 × 10^4^	–	–	+	–	+	+	+
IQE1149	14/F	Iquitos, Loreto	2005 Apr 14	2005 Apr 15	1.0 × 10^3^	–	–	+	–	–	–	+
IQE831	8/F	Iquitos, Loreto	2005 Mar 13	2005 Mar 14	3.0 × 10^2^	–	–	+	–	–	–	+
IQE3605	6/M	Iquitos, Loreto	2006 Apr 3	2006 Apr 4	1.0 × 10^2^	–	–	+	–	+	–	+
IQE3741	10/F	Iquitos, Loreto	2006 Apr 21	2006 Apr 24	5.0 × 10^2^	–	–	+	–	+	–	+
IQE5023	7/M	Iquitos, Loreto	2007 Feb 27	2007 Feb 28	1.3 × 10^4^	–	–	+	–	+	–	+
FMD1737	18/M	Puerto Maldonado, Madre de Dios	2007 Dec 17	2007 Dec 19	5.0 × 10^2^	–	–	+	–	–	–	+
FMD1905	21/F	Puerto Maldonado, Madre de Dios	2008 Feb 9	2008 Feb 12	3.0 × 10^3^	–	–	+	–	+	–	+
Patient 2¶	25/M	Puerto Maldonado, Madre de Dios	2005 Feb 24	2005 Feb 28	NM	+	+	+	–	+	–	+

*Epistaxis, bleeding gums, ecchymosis, purpura, hematochezia, or melena. †Gastrointestinal (nausea, vomiting, diarrhea, or abdominal pain). ‡Respiratory (cough, dyspnea, or cyanosis). §Arthralgia, myalgia, bone pain, malaise, or prostration. ¶Virus isolation attempts unsuccessful; ELISA immunoglobulin M results positive for Venezuelan equine encephalitis. NM, not measured.

Patient 2 was a 25-year-old man from Puerto Maldonado, a city in the department of Madre de Dios (Figure 1). On February 24, 2005, he reported fever, headache, myalgia, nausea, vomiting, and diarrhea. Because he was from an outlying rural area, he was taken to the local health center where he received intravenous rehydration and partially recovered. The patient stayed at home, but his signs and symptoms persisted. On February 27, jaundice and epistaxis developed, and the local health center referred him to the Santa Rosa Hospital in Puerto Maldonado, where he was admitted on February 28. The patient’s status deteriorated quickly; hematuria and hematemesis were followed by liver and renal failure (Table 1), and the patient died on March 1. Postmortem examination found multiple hemorrhages in his lungs, kidneys, and stomach. A serum sample collected at the time of admission was positive for VEEV antibodies, according to ELISA IgM (titer 6,400) (*2*,*3*). The case was presumed to be VEE, although virus isolation attempts were unsuccessful. Serologic assays produced negative results for leptospiral and arboviral diseases, including dengue, Mayaro, yellow fever, Oropouche, and Eastern equine encephalitis.

## Conclusions

Patient 1 had no previous history of neurologic disease or poor health. VEEV subtype ID infection (Panama/Peru genotype) was confirmed. Because her viremia titer was similar to titers of other patients who did not have neurologic complications and survived VEEV infection, viremia levels alone may not account for the difference in disease outcome. Although VEEV was isolated from the patient’s serum and she met the Centers for Disease Control and Prevention’s diagnostic criteria for confirmed VEE (www.cdc.gov/ncphi/disss/nndss/casedef/arboviral_current.htm), we cannot rule out concomitant bacterial meningitis in this patient, who had meningismus and leukocytosis. The limited extent of our diagnostic procedures prevent us from concluding with certainty that VEEV infection was the main cause of death.

Patient 2 had severe hemorrhagic complications. Although an uncommon manifestation of VEEV infection, these complications have been reported elsewhere (*6**,**8**,**14*). To date, only VEEV subtype ID has been isolated in and around Puerto Maldonado (*5*); thus, this patient was probably also infected with this subtype. However, we cannot unequivocally state that the patient died from VEEV infection.

Both fatal cases described in this report were clinically similar to previously reported enzootic and epidemic VEE cases (*1**,**6**,**8**,**9**,**11**,**14*). Initially, both patients had fever, body aches, vomiting, and diarrhea (Table 1) (*1*, *6**,**8**,**9**,**11*), which are also caused by other tropical diseases like dengue. Only some patients, such as patient 1 from Yurimaguas, had neurologic complications (Table 1), which are more commonly observed in children<15 years of age (*6,8,**9**,**11*).

Our surveillance activities were limited to only 8 surveillance sites in Peru (Figure 1), so VEE cases in other areas may remain undiagnosed. Because of the lack of surveillance activities and proper diagnostic capabilities, fatal VEEV infection in Peru is likely; many cases may remain underreported in isolated rural locations where the disease is most common. Additional studies are needed to fully measure the extent and effects of VEE in Peru.
